# Neuroinflammation-Driven Lymphangiogenesis in CNS Diseases

**DOI:** 10.3389/fncel.2021.683676

**Published:** 2021-06-23

**Authors:** Martin Hsu, Collin Laaker, Matyas Sandor, Zsuzsanna Fabry

**Affiliations:** ^1^Neuroscience Training Program, University of Wisconsin Madison, Madison, WI, United States; ^2^Department of Pathology and Laboratory Medicine, University of Wisconsin Madison, Madison, WI, United States

**Keywords:** CNS, meningeal lymphatics, cribriform plate, lymphoid vessels, CNS trauma, CNS autoimmunity, CNS infection

## Abstract

The central nervous system (CNS) undergoes immunosurveillance despite the lack of conventional antigen presenting cells and lymphatic vessels in the CNS parenchyma. Additionally, the CNS is bathed in a cerebrospinal fluid (CSF). CSF is continuously produced, and consequently must continuously clear to maintain fluid homeostasis despite the lack of conventional lymphatics. During neuroinflammation, there is often an accumulation of fluid, antigens, and immune cells to affected areas of the brain parenchyma. Failure to effectively drain these factors may result in edema, prolonged immune response, and adverse clinical outcome as observed in conditions including traumatic brain injury, ischemic and hypoxic brain injury, CNS infection, multiple sclerosis (MS), and brain cancer. Consequently, there has been renewed interest surrounding the expansion of lymphatic vessels adjacent to the CNS which are now thought to be central in regulating the drainage of fluid, cells, and waste out of the CNS. These lymphatic vessels, found at the cribriform plate, dorsal dural meninges, base of the brain, and around the spinal cord have each been implicated to have important roles in various CNS diseases. In this review, we discuss the contribution of meningeal lymphatics to these processes during both steady-state conditions and neuroinflammation, as well as discuss some of the many still unknown aspects regarding the role of meningeal lymphatics in neuroinflammation. Specifically, we focus on the observed phenomenon of lymphangiogenesis by a subset of meningeal lymphatics near the cribriform plate during neuroinflammation, and discuss their potential roles in immunosurveillance, fluid clearance, and access to the CSF and CNS compartments. We propose that manipulating CNS lymphatics may be a new therapeutic way to treat CNS infections, stroke, and autoimmunity.

## Introduction and Significance of Lymphatics

Lymphatic vessels are required for tissue homeostasis, where they typically reside in the tissue parenchyma and serve as a conduit for the clearance of excess, fluid, cells, antigens, nutrients, and waste (Cueni and Detmar, 2008; Oliver et al., 2020; Petrova and Koh, 2020). Consequently, lymphatics maintain tissue homeostasis and provide immune surveillance as soluble antigens along with antigen presenting cells are sampled by the draining lymph nodes through lymphatics. Unlike blood endothelial cells, lymphatic endothelial cells particularly at the capillary level are specialized for the transport of leukocytes; they lack pericyte coverage, often contain discontinuous basement membranes, discontinuous junctions, as well as valves that facilitate uni-directional flow in larger collecting lymphatics (Breslin et al., 2018).

Phylogenetically, the lymphatic system with separate vasculature is believed to first appear in vertebrates. A primitive lymphatic system with evolutionarily conserved structural and cellular features was discovered in zebrafish (Yaniv et al., 2007). Amphibians, reptiles, and flightless birds also have a well-developed lymphatic system with a specialized lymph heart that drives lymph drainage and transport (Hedrick et al., 2013). The lymphatic system further evolved in flying birds and mammals to lose the lymph heart and instead acquire lymph nodes for immune functions. Similar lymphatic drainage was defined across many animal models (Koh et al., 2005; Proulx, 2021), although there are differences between rodent models and humans (Choi et al., 2012). In particular, arachnoid granulations are not found in lower vertebrates or human infants (Proulx, 2021), creating some controversy in CSF drainage pathways in humans. This is reviewed by others (Koh et al., 2005; Proulx, 2021) and briefly discussed later in this review in the context of recent *in vivo* imaging.

Brain lymphatics were shown for the first time in 1787 by Mascagni (Sandrone et al., 2019; Hsu et al., 2021; Proulx, 2021). Since the 1800s, several studies were aimed at characterizing the drainage pathways from the central nervous system (CNS) in which parallels were drawn between the subarachnoid space surrounding the brain and lymphatics in the periphery (Rodriguez-Peralta, 1957; Földi et al., 1966; Andres et al., 1987; Cserr et al., 1992b; Kida et al., 1993; Bozanovic-Sosic et al., 2001; Mollanji et al., 2001b; Koh et al., 2005; Pollay, 2010; Weller et al., 2010; Laman and Weller, 2013; Brøchner et al., 2015; Engelhardt et al., 2017; Hannocks et al., 2018; Castro Dias et al., 2019). While early studies utilized tracer injection into the cerebrospinal fluid (CSF), the discoveries of specific lymphatic endothelial cell markers allowed the description of the fine anatomy of lymphatic vessels surrounding the CNS (Aspelund et al., 2015; Louveau et al., 2015). These markers include the homeodomain transcription factor Prox1, where Prox1 knockout mice fail to develop a proper lymphatic system (Oliver et al., 1993; Wigle and Oliver, 1999; Wigle et al., 2002). The “master control gene for lymphoid vessel development” Prox1 was originally isolated by its protein sequence homology to the Drosophila protein Prospero (Oliver et al., 1993; Tomarev et al., 1996; Zinovieva et al., 1996). Other lymphatic markers include the Lymphatic Vessel Endothelial Hyaluronan Receptor Lyve-1, a homolog of CD44. The function of Lyve-1 by LECs is less understood, since Lyve-1/CD44 double knockout mice still contain a normal lymphatic system without any significant functional defects in tissue fluid homeostasis (Luong et al., 2009). Nevertheless, Lyve-1 has recently been shown to be important for dendritic cell trafficking to the draining lymph nodes through lymphatic vessels during inflammation (Johnson et al., 2017), suggesting that Lyve-1 may contribute more to immune surveillance than fluid homeostasis. Podoplanin, a mucin type transmembrane sialoglycoprotein, is also commonly used to identify lymphatics. Functionally, the lymphatic system of podoplanin knockout mice fail to differentiate from venous endothelial cells, and have been shown to play a role in both lymphatic development and the transmigration of dendritic cells (Bianchi et al., 2017).

## Lymphatic Development, Structure, Markers, and Maturity

Developmentally, lymphatic endothelial cells arise from venous endothelial cells through a complex process involving several signaling molecules and transcription factors that are reviewed by others (Yang and Oliver, 2014; Aspelund et al., 2016; Petrova and Koh, 2020). Briefly, Sox18 can activate the master lymphatic endothelial cell transcription factor Prox1 to promote lymphatic endothelial cell specification, where VEGFC-VEGFR3 signaling is then required to induce sprouting and expansion by lymphatic endothelial cells to promote new vessels (Aspelund et al., 2016). Once developed, lymphatic vessels are structurally divided in capillaries, pre-collectors, and collectors. The lymphatic vessel markers Lyve-1, Podoplanin, CD31, and CCL21 also all play critical roles in the recruitment of leukocytes from the tissue parenchyma (Torzicky et al., 2012; Bianchi et al., 2017; Johnson et al., 2017; Farnsworth et al., 2019). Lymphatic capillaries are enriched for all of these markers and are thus hypothesized to facilitate the majority of exchange between the tissue parenchyma and lymph. Lymphatic capillaries, pre-collectors, and collectors also express Prox1 and VEGFR3 (Yang and Oliver, 2014; Petrova and Koh, 2020), although the capillaries lack valves that facilitate uni-directional flow of lymph. These capillaries then drain into pre-collectors, which contains lower expression of Lyve-1 and CCL21, and structurally contain valves. These then drain into proper collecting lymphatic vessels, which also contains lower expression of CCL21 and Lyve-1, contain valves, and are surrounded by smooth muscle cells.

An extensive network of meningeal lymphatics surrounding the CNS and spinal cord have been identified (Furukawa et al., 2008; Aspelund et al., 2015; Louveau et al., 2015, 2018; Ahn et al., 2019; Hsu et al., 2019; Jacob et al., 2019), which are hypothesized to play significant roles in fluid homeostasis and immune surveillance of the CNS. These lymphatics reside in the meninges, and can be found in the dura above the sinuses on the surface of the brain (Aspelund et al., 2015; Louveau et al., 2015), near the base of the brain (Aspelund et al., 2015; Ahn et al., 2019), near the cribriform plate (Furukawa et al., 2008; Aspelund et al., 2015; Hsu et al., 2019), and surrounding the spinal cord (Aspelund et al., 2015; Jacob et al., 2019). All of these lymphatics have been implicated in draining CSF during steady-state conditions (Table 1), and their dysfunction is associated with many different neuroinflammatory diseases including MS (Louveau et al., 2018; Hsu et al., 2019), glioblastoma (Ma et al., 2019b; Hu et al., 2020; Song et al., 2020), stroke (Yanev et al., 2020), traumatic brain injury (Bolte et al., 2020), cerebrovascular disease (Chen et al., 2019), Parkinson’s disease (Ding et al., 2021), Alzheimer’s disease (Da Mesquita et al., 2018), and aging (Ma et al., 2017; Da Mesquita et al., 2018). Various dyes, macromolecules, and antigens injected into the cisterna magna can be found within each of these meningeal lymphatics networks, although the relative contribution of each of these pathways to fluid drainage is controversial (Table 1). It is unclear how these meningeal lymphatics are able to access the CSF-filled subarachnoid space due to the presence of an arachnoid barrier, an epithelial cell layer containing tight junctions that limits the paracellular permeability of small ions and molecules that separates the subarachnoid space from the dura (Brøchner et al., 2015; Engelhardt et al., 2017; Proulx, 2021). Additionally, it seems as if each meningeal lymphatic network may have differential access to the subarachnoid space.

**TABLE 1:** Summary of CSF drainage pathways in animal models.

References	Species	Tracer	Site of injection	Cervical lymph nodes	Nasal turbinates	Cribriform plate	Dura above the sinuses	Base of the brain	Spinal cord
Yamada et al., 2005	Guinea Pigs	Gadoteridol	Lateral ventricle	–	Yes	Yes	No	–	–
		Gadoteridol	Cisterna magna	–	Yes	Yes	No	–	–
Koh et al., 2005	Non-human Primates	Microfil	Cisterna magna	Yes	Yes	Yes	–	–	–
Nagra et al., 2006	Rats	Human serum albumin	Lateral ventricles	Yes	Yes	Yes	–	–	–
Koh et al., 2006	Rats	Evans blue dye	Cisterna magna	–	Yes	Yes	–	Yes	–
		Microfil	Cisterna magna	–	Yes	Yes	–	Yes	–
	Pigs	Evans blue dye	CSF	–	Yes	Yes	–	Yes	–
		Microfil	CSF	–	Yes	Yes	–	Yes	–
Nagra et al., 2010	Rats	Human serum albumin	Lateral ventricles	–	Yes	Yes	–	–	–
Liu et al., 2012	Rabbits	Microfil	Cisterna magna	Yes	Yes	Yes	–	–	–
Murtha et al., 2014	Rats	Ultravist (for CT Imaging)	Lateral ventricles	Yes	Yes	Yes	No	Yes	Yes
Liu et al., 2012; Aspelund et al., 2015	Mice	PEG-IRDye (Red)	CNS parenchyma (ISF)	Yes	–	Yes	Yes	Yes	–
Louveau et al., 2015	Mice	Qdot 655	Cisterna magna	–	–	–	Yes	–	–
		Evans blue dye	Cisterna magna	–	–	–	Yes	–	–
		Evans blue dye	Intranasal	No	–	–	Yes	–	–
Bedussi et al., 2015	Mice	3 kD Dextran	Striatum	–	No	No	–	–	–
		500 kD Dextran		–	Yes	Yes	–	–	–
		3 kD Dextran	Cisterna magna	–	Yes	Yes	–	–	–
		500 kD Dextran		–	Yes	Yes	–	–	–
Wolf et al., 2016	Rats	Variety of tracers	Intrathecal	Yes	Yes	Yes	Possibly	Yes	Yes
			Cisterna magna						
Ma et al., 2017	Mice	PEG-IRDye680	Lateral ventricle	Yes	–	Yes	No	Possibly	
		PEG-IRDye680CW	Lateral ventricle	Yes	Yes	Yes	–	–	–
		3kDa-AF680	Lateral ventricle	Yes	Yes	Yes	–	–	–
Wolf et al., 2016; Pizzo et al., 2018	Rats	sdAb/IgG	Cisterna magna	Yes	Yes	Yes	Possibly	Possibly	Possibly
Kumar et al., 2018	Rats	Antibodies	Intranasal	Yes	Yes	Yes	–	–	–
Norwood et al., 2019	Mice	Evans blue dye	Cisterna magna	Yes	Yes	Yes	Possibly	Possibly	Possibly
Hsu et al., 2019	Mice	Evans blue dye	Cisterna magna	Yes	Yes	Yes	–	–	–
Jacob et al., 2019	Mice	OVA-AF555	Intrathecal	–	–	–	–	–	Yes
		Lyve-1 Ab	Intrathecal	–	–	–	–	–	Yes
Ma et al., 2019a	Mice	P40D680, evans blue	Lateral ventricle	–	–	–	–	–	Yes
Ahn et al., 2019	Mice	Gadospin P	Cisterna magna	Yes	–	–	No	Yes	–
Guo et al., 2019	Rats	[18F]-PET	Intrathecal	–	–	Yes	No	Yes	Yes
Brady et al., 2020	Mice	IRDye800CW/Evans blue	Cisterna magna	Yes	Yes	Yes	No	No	Yes
		Albumin-AF488							
Rasmussen et al., 2020	Swine	Indocyanine green	Intrathecal	Yes	Yes	Yes	Possibly	Possibly	Yes
He et al., 2020	Rats	Evans blue dye	Lateral ventricle	Yes	Yes	Yes	–	Yes	–
Benveniste et al., 2020	Rats	GD-contrast agent	Cisterna magna	Yes	Yes	Yes	Yes	Yes	Yes

Most lymphatic vessels in mice develop by birth, and once lymphatics have developed VEGFC is no longer needed for lymphatic vessel maintenance (Yang and Oliver, 2014). So far, the only exceptions are intestinal lymphatics (Nurmi et al., 2015) and the dural lymphatics above the CNS, which seem to require sustained VEGFC to maintain baseline levels of lymphatic structure (Antila et al., 2017; Hsu et al., 2019). The meningeal lymphatics above the sinuses develop the latest (P21), and coincidentally require continuous VEGFC production; pharmacological or genetic depletion of VEGFC results in meningeal lymphatics above the sinuses to undergo regression (Antila et al., 2017; Hsu et al., 2019). Interestingly, the requirement of VEGFC to maintain baseline levels is not true for all meningeal lymphatics, as those near the cribriform plate that develop much earlier (P0–P2) (Antila et al., 2017) no longer require VEGFC signaling during steady-state conditions (Hsu et al., 2019). Furthermore, evidence of immaturity by the meningeal lymphatics above the sinuses can be found by sequencing studies, in which genes involved in lymphatic development, proliferation, and stiffness are dysregulated compared to more conventional lymphatics in the periphery (Antila et al., 2017; Louveau et al., 2018). This dysregulation in genes may reflect their significantly delayed development and requirement of continuous VEGFC signaling for maintenance. Additionally, the differences in developmental timeline, maturity, and signaling likely reflects heterogeneity in their function during both steady-state and neuroinflammatory conditions, which will be discussed later in this review.

## Do CNS Lymphatics Have Access to CSF?

Anatomical characterization reveals that the meningeal lymphatics near the base of the brain are in closer proximity to the subarachnoid space than the dural lymphatics above the sinuses when immunolabeling the epithelial cells that make up the arachnoid barrier with E-Cadherin (Ahn et al., 2019), despite still being separated by an uninterrupted arachnoid barrier. In contrast, characterization of the arachnoid barrier in developing rats revealed the lack of an arachnoid barrier separating the cribriform plate from the subarachnoid space when immunolabeling for the arachnoid barrier tight junction protein Claudin-11 (Brøchner et al., 2015), suggesting that cribriform plate lymphatics may have direct access to CSF. Nevertheless, recent studies have highlighted a role for all of these meningeal lymphatics in fluid drainage, with some suggesting that the primary route of drainage is through lymphatics instead of venous routes such as through arachnoid villi or granulations and into dural veins (Ma et al., 2017). For a general overview and historical context for current hypotheses of CSF efflux routes, we cite recent reviews by our lab (Hsu et al., 2021) and/or Steven Proulx (2021). A detailed list of studies investigating CSF efflux routes prior to 2005 is reviewed by Miles Johnston (Koh et al., 2005), and here we summarize more current CSF efflux studies since 2005 (Table 1). In this review, we look to focus on specifically meningeal lymphatics that surround the CNS and review their canonical roles in drainage as well as non-canonical, potentially novel roles in directly regulating immunity through leukocyte cross-talk and regulation.

## Do CNS Lymphatics Have Access to Antigens, Myeloid Cells, and T Cells?

In addition to fluid homeostasis, another primary function of lymphatic vessels is to maintain homeostatic immune surveillance through the drainage of antigens and antigen presenting cells. Although the CNS lacks conventional antigen presenting dendritic cells and lymphatic vessels within the tissue parenchyma, it is able to undergo immunosurveillance even during steady-state conditions (Harris et al., 2014; Rayasam et al., 2018). Several studies have shown that CNS or CSF-derived antigens can be found peripherally when injected (Cserr et al., 1992a,b) or through endogenous expression (Cornet et al., 2001; Cao et al., 2006; Sanchez-Ruiz et al., 2008; Saxena et al., 2008; Schildknecht et al., 2009; Scheikl et al., 2012), in which CNS-derived antigens may be even more immunogenic than when introduced peripherally (Harling-Berg et al., 1989; Gordon et al., 1992; Qing et al., 2000; Ling et al., 2003, 2006; Karman et al., 2004b). When ovalbumin peptides are endogenously expressed in the CNS by crossing an OVA_257–264_-OVA_323–339_-GFP^fl/fl^ transgenic mouse with CNPase-Cre to drive oligodendrocyte expression or Nes-Cre to drive neuronal/glial expression of OVA_257–264_ and OVA_323–339_ peptides, OVA_257–264_-specific CD8 OT-I and OVA_323–339_-specific CD4 OT-II T cell proliferation can be observed in the draining lymph nodes (Harris et al., 2014; Rayasam et al., 2018), suggesting functional and sufficient antigen drainage from the CNS to prime T cells in the draining lymph nodes during steady-state conditions. It’s possible that CNS-derived antigens are able to access meningeal lymphatics as either soluble antigens, or within migratory antigen presenting cells such as dendritic cells. Both OVA-peptides or antigen loaded dendritic cells injected into the CSF can be found near and within meningeal lymphatics. Additionally, functional inhibition of meningeal lymphatic function is sufficient to reduce T cell priming in the draining lymph nodes (Louveau et al., 2018; Hsu et al., 2019), and conversely increasing meningeal lymphatic function through VEGFC can promote immune surveillance of the CNS. These data suggest that the meningeal lymphatics have the capability of draining both soluble and intracellular antigens.

When DQ-OVA, which emits green fluorescence when intact and red fluorescence after proteolytic digestion from being internalized by mononuclear cells, is injected into the CNS, there is an initial wave of soluble antigens that drain to the lymph nodes independently of cells as early as 2 h, followed by a second wave of cell-mediated transport between 8 h and 7 days later (Ling et al., 2003; Hsu et al., 2019). Antigens injected into the CNS parenchyma recruit MHC II^+^ dendritic cells to process and carry the antigen to the draining lymph nodes (Karman et al., 2004b). Furthermore, injection of insoluble antigens into the CNS requires CCR7^+^ dendritic cell emigration (Karman et al., 2004a) where intracellular insoluble antigens can be found within MHC II^+^ dendritic cells near meningeal lymphatics (unpublished), suggesting that the drainage is dependent upon CCL21^+^ lymphatic vessels and CCR7^+^ dendritic cells (Clarkson et al., 2017). Perhaps more interesting is the fact that OVA-GFP peptides can be found within lymphangiogenic vessels near the cribriform plate (unpublished), suggesting their ability to uptake and process antigens which will be discussed in a later section. In contrast, during a mouse model of Alzheimer’s disease it has been reported that CNS-derived amyloid-β can be found accumulating in the dura and near dural lymphatics when dural lymphatics are ablated (Da Mesquita et al., 2018), suggesting that the lymphatics in the dura contribute to drainage of amyloid beta. However, other groups have reported the lack of amyloid-β uptake by the dural meningeal lymphatic endothelial cells (Goodman et al., 2018), suggesting that while they may play a role in facilitating drainage of CNS-derived antigens, they may be unable to uptake and process antigens. Nevertheless, the lymphatics in the dura canonically contribute to the drainage of CSF-derived cells, as they contain significant amounts of antigen presenting dendritic cells which utilize dural lymphatics for drainage to the lymph nodes. It is still unclear if CNS-derived cells are able to access lymphatics in the dura above the sinuses, and if so, how they are able to traverse the arachnoid barrier. It should also be critically noted that although inefficient drainage of amyloid-beta through lymphatics may contribute to the accumulation of amyloid-beta within the CNS, overproduction of amyloid beta within the CNS also likely drives Alzheimer’s disease pathology (Murphy and LeVine, 2010).

Much less is known about how CNS-derived cells may access meningeal lymphatics, especially during steady-state conditions. The majority of data demonstrating the capability of meningeal lymphatics to access antigens and cells focus on access from the CSF compartment by delivery through the cisterna magna (Table 1). Injection of exogenous antigens or cells through the cisterna magna likely disrupts the arachnoid barrier that separates the CSF-filled subarachnoid space from the meningeal lymphatics, which may lead to ambiguous interpretations about the extent of meningeal lymphatic access to the subarachnoid space. Additionally, drainage of migratory antigen presenting cells from the CNS is relatively rare due to the lack of conventional dendritic cells within the CNS during steady-state conditions, and is confounded by the fact that the meninges contain a large number of conventional dendritic cells (Mrdjen et al., 2018; Van Hove et al., 2019; Rustenhoven et al., 2021). During neuroinflammation however, there is a large accumulation of dendritic cells into the CNS (Engelhardt et al., 2001; Mohammad et al., 2014; Clarkson et al., 2015; Ge et al., 2017; Schiefenhövel et al., 2017) followed by drainage of antigens and dendritic cells to the draining lymph nodes (Mohammad et al., 2014). Therefore, studying antigen and cell trafficking from the CNS to the draining lymph nodes through meningeal lymphatics may require neuroinflammation as a model to study. CNS-derived antigens can be found within lymphangiogenic vessels near the cribriform plate as well as dural lymphatics above the sinuses, although how CNS-derived antigens or cells migrate from the CNS to the meningeal lymphatics remains unclear.

Our laboratory has previously shown using a photoconvertible KikGR transgenic mouse that photoconverted cells in the CNS can in fact be found in meningeal lymphatics near the cribriform plate during neuroinflammation and consequently the draining lymph nodes (Hsu et al., 2019), suggesting that a subset of CNS-derived cells utilize cribriform plate lymphatics to exit the CNS. A similar experiment (Louveau et al., 2018) was performed to track meningeal-derived T cells and/or dendritic cells in the subarachnoid space, pia, and/or dura to the deep cervical lymph nodes, suggesting that the dural lymphatics above the sinuses connect to CNS-draining lymph nodes. In these experiments, intracerebral or meningeal photoconversion likely recruits additional inflammatory cells to the photoconversion site to increase the number of photoconverted cells and thus increase the odds of tracking photoconverted cell migration. However, it is also possible that disruption of the arachnoid barrier from the photoconversion contributed to additional cells being found within meningeal lymphatics and the draining lymph nodes. Future studies are needed to elucidate the ability and extent of the different meningeal lymphatic networks in the drainage of CNS-derived cells without disrupting the arachnoid barrier, especially since how meningeal lymphatics are able to access the subarachnoid space through the arachnoid barrier remains unknown.

As mentioned in a previous section, an arachnoid barrier separates the CSF from the lymphatic vessels that reside in the dura surrounding the CNS (Engelhardt et al., 2017; Hannocks et al., 2018) with the exception for the cribriform plate, which seems to lack E-Cadherin and Claudin-11 expression (Brøchner et al., 2015). Interestingly, several independent cell trafficking studies using experimental autoimmune encephalomyelitis (EAE) as a model of neuroinflammation have tracked dendritic cell influx through the choroid plexus where they migrate rostrally toward the olfactory bulbs near the cribriform plate along the rostral migratory stream across disease progression (Clarkson et al., 2015; Schiefenhövel et al., 2017). Functionally, inhibition of dendritic cell migration along the rostral migratory stream during EAE reduces dendritic cell drainage to the draining lymph nodes (Mohammad et al., 2014), suggesting that migration from the choroid plexus toward the olfactory bulbs near the cribriform plate contribute significantly to CNS-derived cell drainage. Once a dendritic cell has reached the olfactory bulbs near the cribriform plate, it is unclear how they are able to exit the CNS parenchyma to traverse the CSF and gain access to the meningeal lymphatics near the cribriform plate, even with the lack of an arachnoid barrier. As mentioned previously, the arachnoid barrier seems to have gaps where the cribriform plate lymphatics reside (Brøchner et al., 2015), and tracers infused into the CSF flow rostrally toward the cribriform plate (Table 1), suggesting that meningeal lymphatics in this region are in a prime position to sample CSF. Alternatively, a dendritic cell theoretically does not need to leave the CNS parenchyma to traverse along the perineural sheaths of the olfactory cranial nerves, as these neurons send axons from the olfactory submucosa through the cribriform plate to synapse directly with neurons in the olfactory bulb. However, if and when this happens, it is unclear how a dendritic cell exits the perineural space to migrate toward lymphatics in the nasal submucosa.

In addition to T cells and dendritic cells, other leukocytes such as macrophages have been observed in proximity to meningeal lymphatics during neuroinflammation (Hsu et al., 2019; Rustenhoven et al., 2021), perhaps suggesting macrophage recruitment by meningeal lymphatics. A subset of macrophages can serve as professional antigen presenting cells such as those in the dura, suggesting a potential role for their ability to capture and present antigen to T cells (Rustenhoven et al., 2021). Similar to dendritic cells, macrophages have also been shown to produce VEGFC to drive VEGFR3-dependent lymphangiogenesis near the cribriform plate (Hsu et al., 2019), suggesting that the signaling cues that recruit VEGFC-producing dendritic cells to the cribriform plate may be shared with other myeloid cells such as macrophages. Furthermore, macrophages have been shown to directly contribute to lymphangiogenesis by transdifferentiation into lymphatic endothelial cells during inflammation (Volk-Draper et al., 2017; Ran and Volk-Draper, 2020). However, whether this occurs in any of the meningeal lymphatic networks during steady-state or neuroinflammatory conditions is unknown.

## Lymphangiogenesis by Meningeal Lymphatics

Heterogeneity and plasticity are two remarkable features of lymphatic endothelial cells (Lee et al., 2010). Genome-wide transcriptional profiling of human dermal blood endothelial cells vs. lymphatic endothelial cells revealed that more than 95% of genes are comparatively expressed by the two endothelial cell types (Podgrabinska et al., 2002; Hirakawa et al., 2003). However, LEC identities appear to be highly plastic, reversible, and heterogenous. Single cell RNA sequencing of human lymph nodes reveal several subtypes of lymphatic endothelial cells with unique specialized functions such as leukocyte migration, antigen presentation, pathogen interactions, cell-cell interactions, and tolerance (Takeda et al., 2019; Xiang et al., 2020). Additionally, lymphangiogenesis is common during inflammation, and can be seen for example in cancers (Christiansen and Detmar, 2011), *Mycobacterium tuberculosis* infections (Harding et al., 2015), skin inflammation (Varricchi et al., 2015), ocular inflammation (Chauhan et al., 2014), along with many other models of inflammation and is even hypothesized to facilitate immune memory for subsequent infections (Kelley et al., 2013; Chauhan et al., 2014; Kim et al., 2014).

Heterogeneity can also be observed between the different meningeal lymphatic networks in the dura and cribriform plate in both steady-state conditions as well as different models of neuroinflammation. As mentioned previously, the earlier development of meningeal lymphatics near the cribriform plate (Antila et al., 2017) coincides with their stability of no longer requiring VEGFR3 signaling to maintain baseline levels, as pharmacological inhibition of VEGFR3 signaling using MAZ51 in adult mice does not affect cribriform plate meningeal lymphatic morphology (Hsu et al., 2019). In contrast, the lymphatics residing in the dura develop several weeks after birth in mice (Antila et al., 2017), and consequently require continuous VEGFR3 signaling to maintain baseline levels. While lymphangiogenesis is unique to the cribriform plate during EAE, heterogeneity in the lymphangiogenic capability of different meningeal lymphatic networks can also be seen in different models of neuroinflammation. Similar to EAE, there is also a lack of lymphangiogenesis by dural lymphatics in a transient middle cerebral artery occlusion model of stroke (Yanev et al., 2020) despite elevated levels of VEGF in this model (Cosky and Ding, 2018; Yanev et al., 2020). However, lymphangiogenesis by dural lymphatics can be observed in other models of neuroinflammation including a photothrombotic model of stroke, a model of brain tumor where tumor cells are injected subdurally (Hu et al., 2020), traumatic brain injury (Bolte et al., 2020), cranial EEG electrodes, and during cranial window preparations (Hauglund et al., 2020). These studies suggest that lymphangiogenesis by different meningeal lymphatic networks may depend upon the model of neuroinflammation.

The cellular source(s) of VEGFC in the dura and near cribriform plate meningeal lymphatics may be different, which may explain why lymphangiogenesis during EAE is unique to cribriform plate lymphatics. During steady-state conditions, smooth muscle cells provide enough VEGFC to maintain baseline levels of lymphatics in the dura (Antila et al., 2017), while the meningeal lymphatics near the cribriform plate recruit VEGFC producing myeloid cells to mediate VEGFR3-dependent lymphangiogenesis during neuroinflammation (Hsu et al., 2019). During EAE, a relatively large increase in myeloid cells can be found near the cribriform plate, which may partially be explained by the observation that there is relatively little myeloid cell numbers near the cribriform plate during steady state. In contrast, the dura is enriched with resident macrophages and dendritic cells during steady-state conditions. Therefore, while the dural lymphatics and cribriform plate lymphatics are able to produce CCL21 during neuroinflammation to recruit CCR7^+^ leukocytes (Louveau et al., 2018; Hsu et al., 2019), cribriform plate lymphatics may uniquely undergo lymphangiogenesis during EAE due to the relatively large increase in VEGFC-producing myeloid cells compared to steady-state. Although myeloid cells in the dura have different phenotypes between steady-state and neuroinflammation (Rustenhoven et al., 2021), it is unknown if the myeloid cells or any other cell in the dura are able to produce increased levels of VEGFC during neuroinflammation.

Access to the CNS and CSF compartment may also be different between the different sites of meningeal lymphatics. Anatomical studies in developing rats revealed the lack of an arachnoid barrier near the cribriform plate (Weller et al., 2018), suggesting that meningeal lymphatics near the cribriform plate may have direct, unrestricted access to the subarachnoid space where pro-lymphangiogenic factors such as VEGFC may be present during neuroinflammation. There is abundant evidence of CSF access and sampling by meningeal lymphatics in the dura above the sinuses (Table 1), however, several studies have revealed that the arachnoid barrier seems to remain in-tact in arachnoid villi (Alksne and White, 1965; Shabo and Maxwell, 1968; Alksne and Lovings, 1972), suggesting that access by meningeal lymphatics in the dura to the subarachnoid space may be somewhat limited (Ma et al., 2017; Ahn et al., 2019). Therefore, although the dural meningeal lymphatics above the sinuses can clearly sample CSF, how they access CSF through the arachnoid barrier is currently unknown (Proulx, 2021).

Another theory is that the amount of VEGFC needed for lymphangiogenesis by the different meningeal lymphatic networks may be different. Although the meningeal lymphatics in the dura above the sinuses can undergo lymphangiogenesis in the presence of AAV-VEGFC (Antila et al., 2017; Bolte et al., 2020; Song et al., 2020), the amount of VEGFC delivered through AAV likely exceeds endogenous VEGFC levels even during neuroinflammation. Indeed, heterogeneity in the stability of the meningeal lymphatics can be seen in steady-state conditions when mice are treated with the VEGFR3-tyrosine kinase inhibitor MAZ51. Treatment of naive adult mice with MAZ51 causes the meningeal lymphatics in the dura above the sinuses to undergo regression, while those near the cribriform plate are unaffected (Hsu et al., 2019). This likely has to do with the developmental maturity of these lymphatic networks, where lymphatics such as those near the cribriform plate that have developed by postnatal day 2 no longer require sustained VEGFC-VEGFR3 signaling for maintenance. In contrast, the meningeal lymphatics in the dura above the sinuses develop quite late (P21 in mice) (Antila et al., 2017), which may also explain their unique phenotype when compared to other peripheral lymphatics.

It is also important to note that meningeal lymphatics can also be found at the base of the brain and spinal cord (Aspelund et al., 2015; Ahn et al., 2019; Jacob et al., 2019), and that the dural lymphatics above the sinuses can extend laterally along the transverse sinuses for quite a distance (Aspelund et al., 2015; Louveau et al., 2018). This means that the meningeal lymphatics surrounding the CNS make up quite a large network, and it is unclear if they remain as separate networks or are all connected. Although the lymphatics near the base of the brain have also been implicated in draining CSF (Ahn et al., 2019), it is unclear if they are able to undergo lymphangiogenesis during neuroinflammation. The anatomical location of the basal meningeal lymphatics have made it difficult to study, and future studies are needed to fully elucidate other networks of meningeal lymphatics surrounding the brain during both steady-state and neuroinflammation, as well as if any of these described lymphatics are connected.

Notably, lymphangiogenesis within CNS-draining lymph nodes has also been reported during reperfusion in a mouse model of stroke (Esposito et al., 2019). Intriguingly, lymphangiogenesis in the cervical lymph nodes could be observed as early as 24 h after stroke when measuring Podoplanin and Lyve-1, however, whether or not lymphangiogenesis can occur this quickly remains controversial. Meningeal lymphatics near the cribriform plate require several days to undergo lymphangiogenesis during EAE (Louveau et al., 2018), suggesting that either the draining lymph node lymphatics are able to undergo unprecedented rates of lymphangiogenesis in response to neuroinflammation, or non-lymphatic endothelial cells in the draining lymph nodes express lymphatic markers during neuroinflammation. Indeed, Podoplanin has been reported to be expressed by a subset of CD4 T cells during EAE (Peters et al., 2015), and Lyve-1 can be expressed by a subset of macrophages (Lim et al., 2018; Chakarov et al., 2019; Brezovakova and Jadhav, 2020).

## Potential Functions of Lymphangiogenic Vessels

Fluid accumulation and swelling often accompanies inflammation, which in the case of neuroinflammation may be life-threatening due to the non-expansive nature of the skull. We have reported that lymphangiogenesis by vessels near the cribriform plate serves to clear excess fluid, cells, and antigens from the CSF and CNS to maintain homeostasis and promote recovery during later stages of EAE (Hsu et al., 2019). Lymphangiogenesis near the cribriform plate doesn’t occur until after EAE onset, leukocyte infiltration, and demyelination (Louveau et al., 2018; Hsu et al., 2019), suggesting that the role of lymphangiogenesis likely revolves around the later phase of EAE. Interestingly, pharmacological inhibition of lymphatic function prior to EAE onset using the VEGFR3 tyrosine kinase inhibitor MAZ51 results in decreased EAE severity and delayed EAE onset (Hsu et al., 2019), likely by inhibiting the function of dural lymphatics by causing their regression. However, administration of MAZ51 after the onset of EAE when active lymphangiogenesis by the cribriform plate is occurring results in no change in EAE clinical scores (Hsu et al., 2019), suggesting that there may be opposing roles of the dural lymphatic vessels above the sinuses and meningeal lymphatics near the cribriform plate.

Recently, relatively novel roles have been reported by lymphatic endothelial cells in the lymph node during inflammation. These include antigen processing, antigen archival, antigen presentation, and tolerance induction (Rouhani et al., 2015; Humbert et al., 2016; Yeo and Angeli, 2017; Lucas and Tamburini, 2019; Santambrogio et al., 2019), suggesting that lymphatic endothelial cells may play a more direct role in regulating immunity during inflammation than previously appreciated by acting as non-professional antigen presenting cells. So far, these novel functions have been restricted to lymphatic endothelial cells of the lymph nodes, and it remains to be seen whether any of the meningeal lymphatics are capable of any of these functions. Characterization of the dural tissues have identified antigen presenting cells, MHCII^+^ macrophages and conventional dendritic cells, in the dura in close proximity to dural lymphatics that can uptake CSF-delivered antigens to recruit and present to antigen specific CD4 T cells during steady-state conditions, with no significant changes in antigen presenting cell identity or numbers during neuroinflammation (Rustenhoven et al., 2021). Interestingly, lymphatics in the dura seemed to lack the ability to intracellularly uptake CSF-delivered antigens and engage in antigen archival/presentation, although they seemed sufficient to engage in their canonical role in facilitating leukocyte drainage, including mediating antigen-containing dendritic cell trafficking. Consequently, therapeutically targeting meningeal lymphatic drainage has generated interest in many models of neuroinflammation including MS (Louveau et al., 2018; Hsu et al., 2019), glioblastoma (Ma et al., 2019b; Hu et al., 2020; Song et al., 2020), stroke (Yanev et al., 2020), traumatic brain injury (Bolte et al., 2020), cerebrovascular disease (Chen et al., 2019), Parkinson’s disease (Ding et al., 2021), Alzheimer’s disease (Da Mesquita et al., 2018), and aging (Ma et al., 2017; Da Mesquita et al., 2018), suggesting that at least some of the pathologies associated with these diseases can be attributed to improper meningeal lymphatic function.

**FIGURE 1:**
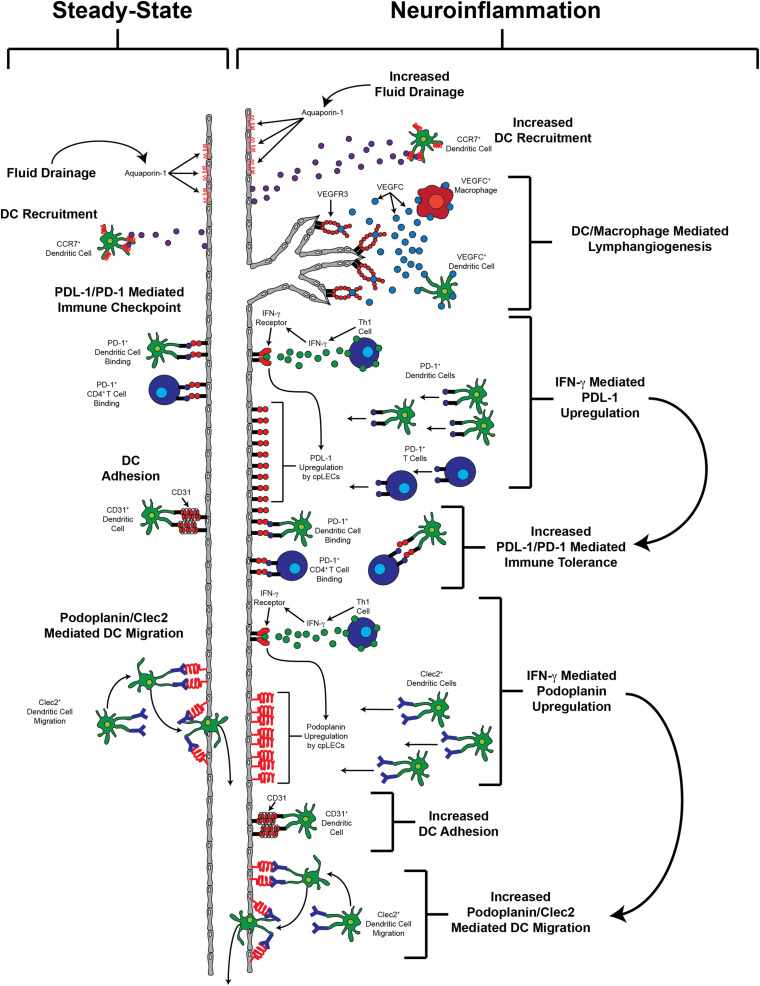
Potential functions of lymphangiogenic vessels.

While proper meningeal lymphatic drainage of fluid, cells, and antigens have generated interest as a therapeutic target, we hypothesize that meningeal lymphatics that undergo neuroinflammation-induced lymphangiogenesis may play a more significant role in neuroinflammation by directly engaging in leukocyte crosstalk (Figure 1), similar to recent evidence identifying novel functions of LECs in the lymph nodes (Takeda et al., 2019; Xiang et al., 2020). As mentioned previously, neuroinflammation-induced lymphangiogenesis by different meningeal lymphatic networks can be observed in different models of neuroinflammation, where post-lymphangiogenic vessels likely contain a unique phenotypic signature compared to steady-state meningeal lymphatic vessels. Indeed, a recent study using bulk RNA sequencing have shown enrichment of genes involved in antigen processing and presentation by lymphangiogenic vessels in the dura above the sinuses after subdural injection of tumor cells (Hu et al., 2020), suggesting that meningeal lymphatics may have the ability to acquire the antigen processing and presentation machinery during after neuroinflammation-induced lymphangiogenesis to directly regulate immunity. However, further studies are needed to fully elucidate the extent, kinetics, and significance of leukocyte crosstalk with lymphangiogenic lymphatic endothelial cells in the meninges during CNS autoimmunity, and whether or not meningeal lymphatics can functionally engage in antigen presentation.

## Lymphangiogenesis: Regression, Maintenance, and Memory

Inflammatory lymphangiogenesis around the brain is often studied in unidirectional path, with the prevailing insight that meningeal lymphatics can adaptively expand to compensate for the increased biological needs of neuroinflammation. Frequently overlooked however, is the process of maintenance or regression of lymphatic vessels after the initial inflammatory lymphangiogenesis has occurred and how these vessels dynamically change across an organism’s lifespan in response to each inflammatory challenge. Interestingly, while it is known that meningeal lymphatics can acquire dysfunctional profiles with age which are associated with altered immune related expression and reduced drainage ability (Ma et al., 2017; Da Mesquita et al., 2018), in the context of neuroinflammation there is little known about the long-term fate of these lymphatic vessels after the resolution of neuropathology and only a few studies have extended their investigation past initial pathology. For example in a mouse model of TBI, dural lymphatic vessel area, diameter, and sprouting returned to baseline levels approximately 1 month post brain injury indicating a mechanism of meningeal lymphatic regression. These vessels were also associated with reduced drainage ability, as well as a morphological phenotype of sustained lymphatic “loops,” 2 months after injury indicating some form of persistent vascular complexity and dysfunction (Bolte et al., 2020).

In the periphery the long-term fate of lymphatic vessels after lymphangiogenesis is also not completely understood and data is conflicting. One common observation is the presence of lymphatic hyperplasia after sustained lymphangiogenic stimulation from VEGF-C (Yao et al., 2014). For example, VEGF-C stimulation for 1–2 weeks on adult mouse skin has been shown to result in lymphatic hyperplasia that persists up to 6 months post stimulation (Lohela et al., 2008). In an animal model of airway inflammation, non-regressing pulmonary lymphatics were reported to persist for months after resolution of inflammation (Baluk et al., 2005). Additionally our lab has shown that in a model of murine BCG liver infection that lymphangiogenesis continues for up to 10 weeks despite resolution of the initial infection (Harding et al., 2015). Other studies in the periphery, however, have shown marketed regressions of lymphatic structures after the resolution of inflammation. Of note, researchers showed that in a model of short-term cornea inflammation, lymphatic growth was reported to have peaked at day 14, followed by a decline to the virtually non-existent lymphatic baseline level 6-months later (Cursiefen et al., 2006) and may be associated with reduced leukocyte trafficking ability and morphology (Kelley et al., 2011). Interestingly, like the brain, the eye exists with an immune privileged status characterized by the absence of infiltrating lymphatics and a tightly controlled immune cell barrier, so similar parallels may exist between the two physiological systems (Wekerle and Sun, 2010). In the lymph nodes, one study indicated that the presence of INF-γ secreting T-cells may act as negative regulators of lymphatic vessel expansion (Kataru et al., 2011), which may potentially highlight a role in pruning lymphatic vessels outside of nodes, although absence of inflammation induced VEGF-C secretion appears to be the main culprit driving post-inflammatory meningeal lymphatic maintenance and regression as evidenced by its inhibition in a number of studies (Antila et al., 2017; Hsu et al., 2019).

An important consideration is the idea of lymphatic vessel memory upon the resolution and experience of neuropathology. There is some evidence that shows that after initial insult and regression, lymphatic vessels in the periphery acquire the ability to quickly initiate the growth kinetics of lymphangiogenesis in response to secondary inflammatory stimuli. Interestingly this pre-priming of lymphatic vessels to future inflammation is thought to occur independently of VEGF-C signaling and is perhaps controlled by surrounding immune cells (Kelley et al., 2013; Chauhan et al., 2014; Kim et al., 2014). While this phenomenon has been scarcely studied in models of neuroinflammation, a rapid lymphangiogenic phenotype may be especially relevant in diseases of periodic or irregular neuroinflammation such as relapse-remitting multiple sclerosis (MS) or a secondary traumatic brain injury, both of which have sustained recovery times between secondary neuroinflammatory stimuli. Finally it should be noted that many neuroinflammatory diseases, including those mentioned previously, never resolve and are associated with sustained chronic inflammatory states within the brain, as seen in conditions like Alzheimer’s disease (Akiyama et al., 2000). These chronic inflammatory diseases are inevitably associated with a multi-year relationship between neuroinflammatory signals, cells, and meningeal lymphatic structure and function. Together these studies indicate that the long-term post-inflammatory fates of meningeal lymph vessels may be equally as important to the initial lymphangiogenic expansion, and that there is need for extended time-course studies on the function of meningeal lymphatic vessels in the context of chronic or irregular neuroinflammation across an organism’s lifespan.

## Comparing Animal Models With Human Studies

An extensive number of studies characterizing routes of CSF drainage done in mice, rats, cats, dogs, pigs, sheep, non-human primates, and humans revealed relatively conserved drainage pathways across species near the cribriform plate. An overwhelming majority of studies, post-mortem and live-imaging, in both animal models (Table 1) and humans (Table 2) reveal an accumulation of CSF on the CNS side of the cribriform plate, where the cribriform plate meningeal lymphatics reside. Lymphangiogenesis by these particular lymphatics are also restricted to the CNS-side of the cribriform plate, confirming their ability to access pro-lymphangiogenic factors from the CNS during neuroinflammation. Microscopic studies using the lymphatic capillary marker Lyve-1 reveal that these cribriform plate meningeal lymphatics can in fact cross the cribriform plate and into the middle nasal turbinate (Hsu et al., 2019). However, once they cross the cribriform plate they lose expression of Lyve-1 in the lamina propria, suggesting their transition out of capillaries and into collecting lymphatics which remain uncharacterized. Vessels can also be observed basal to the brain connecting the cribriform plate to the base of the brain, and the meningeal lymphatics that reside on the CNS side of the cribriform plate can be found throughout the base of the olfactory bulbs. Therefore, it may also be possible that in addition to crossing the cribriform plate, a subset of the lymphatics in this region may also remain on the CNS side of the skull and exit with the jugular foramen along with the meningeal lymphatics at the base of the brain.

**TABLE 2:** Summary of *in vivo* CSF imaging evidence in humans.

References	Imaging technique	Tracer and site of injection	Patient population	Drainage route(s) identified
Ringstad and Eide, 2020	MRI	Intrathecal gadobutrol	18 patients with a variety of CSF disorders	Parasagittal Dura: 18/18
				Base of the brain: 17/18
				Cribriform plate: 2/18
Melin et al., 2020	MRI	Intrathecal gadobutrol	24 patients with a variety of CSF disorders	Strong accumulation of tracer on the CNS side of the cribriform plate, tracer drainage in the inferior turbinates in 11/24 patients, none in nasal mucosa
Kuo et al., 2018	MRI	No tracer	6 healthy patients	Dural lymphatic flows opposite to venous sinuses and directed toward the cribriform plate
de Leon et al., 2017	PET	Intravenous 18F-THK5117	8 Alzheimer’s patients and 7 normal controls	CSF egress through the cribriform plate and into nasal turbinates in all patients; AD patients have reduced drainage through the cribriform plate
		11C-Cocaine	4 normal patients	
Absinta et al., 2017	MRI	Intravenous gadobutrol	5 health volunteers	Accumulation of tracer in the dural lymphatics, middle meningeal artery, and cribriform plate

Tracers infused into the CSF can be observed flowing through the cribriform plate in relatively large quantities in many animal models, and it is also important to note that drainage through the cribriform plate may also occur through olfactory cranial nerves and into downstream lymphatics (Johnston et al., 2004; Koh et al., 2005). In addition to meningeal lymphatics on the CNS side of the cribriform plate, lymphatics can also be observed near the cribriform plate on the nasal mucosa side, as well as more distally in the nasal mucosa (Louveau et al., 2018). It is unknown if the lymphatics on both sides of the cribriform plate are connected, or whether perineural drainage along the olfactory cranial nerves that exit the cribriform plate independently of meningeal lymphatics near the cribriform plate occurs followed by drainage into nasal mucosal lymphatics either near the cribriform plate or further downstream (Koh et al., 2005; Proulx, 2021). In mice where the relative surface area of the cribriform plate is larger relative to humans, dyes can be observed in the nasal mucosa parenchyma, suggesting CSF physically crosses the cribriform plate in large quantities (Johnston et al., 2004; Koh et al., 2005; Ma et al., 2017). Functionally, sealing the cribriform plate in sheep is sufficient to increase intracranial pressure (Mollanji et al., 2001a; Papaiconomou et al., 2002), implying that CSF drainage occurs at least partially through the cribriform plate either along olfactory cranial nerves and into nasal mucosal lymphatics, or within meningeal lymphatic capillaries on the CNS side of the cribriform plate that drain through the cribriform plate into lymphatic collectors in the nasal mucosa. In contrast, pharmacological inhibition of specifically olfactory sensory axons does not alter intracranial pressures in mice (Norwood et al., 2019), suggesting that drainage through the cribriform plate can occur independently of olfactory sensory axons and likely within meningeal lymphatics that cross the cribriform plate.

As mentioned above, recent live imaging studies in humans have generated some discrepancy for the pathway of extracranial nasal lymphatics, which may or may not be connected to meningeal lymphatics on the CNS side of the cribriform plate. In the majority of these imaging studies in humans, CSF can be found on both the CNS side of the cribriform plate where the meningeal lymphatics reside and in the immediate vicinity of the nasal side of the cribriform plate (Table 2), with the discrepancy being how much CSF makes its way distally to the nasal lymphatics in the nasal mucosa. This suggests that if extracranial lymphatics in the nasal mucosa were to contribute to the drainage of CSF in humans, they would need to reside quite close to the cribriform plate. In contrast, in animal models dyes infused into the cisterna magna can flow through the cribriform plate and be found quite distally in the nasal mucosa away from the cribriform plate when viewed sagittally. Interestingly, two recent reports using MRI in humans revealed the lack of CSF efflux through the cribriform plate into the nasal epithelium, despite the accumulation of CSF on the CNS side of the cribriform plate where the meningeal lymphatics reside (Melin et al., 2020; Ringstad and Eide, 2020). Of note, these studies were done in patients that had intracranial hypertension, which may explain some of the confounding results between human studies. Indeed, altering CSF dynamics using a mathematical modeling approach reveals reduced CSF efflux through the cribriform plate after infusion of CSF to increase intracranial pressure. In line with these studies, reduced CSF absorption has been observed in patients with idiopathic intracranial hypertension (Orefice et al., 1992). Alternatively, alterations in CSF dynamics may also skew the preference of CSF toward one route or another (Boulton et al., 1998; Norwood et al., 2019). This may consequently explain the lack of tracer outside of the cribriform plate in these studies. Another issue may be resolution, where diffuse CSF in the subarachnoid spaces on the CNS side of the cribriform plate and diffusion into the dural parenchyma can be observed in the average 1 mm^3^ voxel size resolution of an average 3T MRI machine (Ringstad and Eide, 2020). However, as CSF becomes intravascularized within lymphatic capillaries where the average diameter ranges from 10–60 μm, which then drain into lymphatic collectors that have an average diameter of 0.2 mm (Margaris and Black, 2012), the resolution of a 1 mm^3^ MRI voxel size may be a limiting factor. Furthermore when using PET imaging after tracer infusion in Alzheimer’s disease patients, CSF can in fact be observed outside of the cribriform plate and is reduced compared to healthy controls (de Leon et al., 2017). Although the resolution of PET imaging is inferior to MRI, the use of tracers allow for more sensitive measurements, suggesting that multiple imaging modalities may be needed to fully elucidate the drainage routes in humans. Live imaging studies in human patients remain lackluster compared to animal models (Tables 1, 2), and advances in neuroimaging techniques using multiple modalities will provide clarity in CSF drainage routes in humans.

## Conclusion

Research into the role of meningeal lymphatics in CNS diseases has gained significant attention. The promise that modulating meningeal lymphatics to beneficially impact therapies of CNS diseases justifies this interest. Here we discussed that CNS drainage is crucial in the resolution of neuroinflammatory diseases of the brain, and although multiple pathways might contribute to CNS meningeal lymphatic drainage, the cribriform plate-mediated drainage might have a special role. This is due to (1) the location and unique access of cribriform plate lymphatics to CSF; (2) the adaptability of cribriform plate lymphatics to respond to neuroinflammation by rapid lymph angiogenesis; and (3) the phenotypic adaptation of cribriform plate lymphatic to crosstalk with immune cells and induce an immunomodulatory niche within the meningeal lymphatics. Future research using multiple modalities involving imaging, anatomical, functional and phenotypic studies will be important to understand the full potential for cribriform meningeal lymphatic therapies in CNS disease.

## Author Contributions

MH, CL, MS, and ZF wrote the manuscript. MH generated the figure and tables. All authors edited the manuscript.

## Conflict of Interest

The authors declare that the research was conducted in the absence of any commercial or financial relationships that could be construed as a potential conflict of interest.
